# DNA Methylation Changes Reflect Aluminum Stress in Triticale and Epigenetic Control of the Trait

**DOI:** 10.3390/ijms26114995

**Published:** 2025-05-22

**Authors:** Agnieszka Niedziela, Renata Orłowska, Piotr Tomasz Bednarek

**Affiliations:** Plant Breeding and Acclimatization Institute—National Research Institute, Radzików, 05-870 Błonie, Poland; a.niedziela@ihar.edu.pl (A.N.); r.orlowska@ihar.edu.pl (R.O.)

**Keywords:** triticale, aluminum stress, epigenetic alterations, DNA methylation

## Abstract

Aluminum (Al) stress is typical for acidic soils and may affect cereals’ yield. Al tolerance in triticale is mostly affected by the aluminum-activated malate transporter (ALMT) gene (7R) and some other QTLs (3R, 5R, and 6R). The trait is heritable in about 36% of cases, indicating that epigenetic factors may impact the phenomenon. This study demonstrates that utilizing different methods to quantify DNA methylation changes induced by Al stress results in detail differences, and the results evaluated should be compared critically. The Common and the basic General approaches are sufficient if general information is needed. The General (extended variant) approach may deliver data on methylation changes affecting symmetric sequence contexts. The markers assigned to DN-CG, DM-CG, and DN-CHG were suggested as the most important in explaining Al tolerance in triticale. Analysis of the maps constructed based on root tips and leaf tissues showed different densities of the epigenetic markers but reflected the comparable patterns of their distribution, supporting the hypothesis that Al stress could be transmitted to other plant tissues due to somatic memory. Methylation changes occur throughout the genome and are not associated with specific genes related to aluminum stress.

## 1. Introduction

Aluminum (Al) toxicity is a significant abiotic stress affecting plant growth and productivity in over 50% of the world’s arable land where soil acidity is a problem [1]. In acidic soils (pH < 5.5), aluminum is usually present in its soluble Al^3+^ ions, which are readily available for uptake by plant roots [2]. Al^3+^ ions absorbed in roots inhibit cell division and elongation, disrupt nutrient uptake, and lead to growth cessation [3]. 

Triticale (x *Triticosecale* Wittmack, AABBRR, 2n = 6x = 42), a man-made hybrid between wheat (*Triticum* ssp.) and rye (*Secale* ssp.), was developed to inherit the desirable qualities of both parents, making it an adaptable and resilient crop. Its energy-rich grain with high protein content and higher amount of lysine content compared to other cereals makes it a valuable component of animal diets [4]. While its primary application is in feeding animals, triticale is also used in human food production for bread, pasta, and cereals [4], and in industrial applications like biodegradable films [5,6] and bioethanol production due to its high starch content [7,8]. Triticale has emerged as a model species for studying aluminum tolerance, particularly due to its diverse genotypic responses to Al stress [9,10,11]. However, understanding the genetic and epigenetic mechanisms underlying Al tolerance in triticale is far from complete [12,13,14].

As a complex trait, Al tolerance is affected by many genes, and different mechanisms are triggered to mitigate the toxic effects of the metal. The most recognized is the exudation of organic acids (OA) (i.e., as malate or citrate) from the root apex [2,3]. These acids are transported from the root cells into the rhizosphere, where they bind to Al^3+^ ions, forming non-toxic complexes that are less likely to enter the root. Organic acids distribution is facilitated by specific transporters, mostly belonging to the aluminum-activated malate transporters (ALMT) [15,16,17] and multidrug and toxin efflux (MATE) families [15,18,19]. The genes coding ALMT and MATE proteins have been mapped to chromosomes 3R, 4R, 6R, and 7R in rye [20,21,22,23]. Similarly, Al-tolerant genes were assigned to 2H, 3H, and 4H in barley [24,25] and to 4D, 4B, and 3B in wheat [15,18,19]. Regarding triticale, the ALMT gene encodes a malate transporter, which plays a critical role in Al^3+^ detoxification and is located in the QTL region on the 7R chromosome and explains 36% of phenotypic variance [26].

In parallel to genetic aspects of Al tolerance, epigenetic input, defined as heritable changes in gene expression without DNA sequence changes [27], regarding abiotic stresses, is discussed [28,29,30]. Such changes, among others, could be reflected at the histone modification level [31,32] or as DNA methylation alterations [33]. The latter affects symmetric (CG and CHG) or asymmetric (CHH) sequence contexts, influencing chromatin structure and transcription [33,34].

It was also evidenced that transposable elements (TEs) methylation in or near genes influenced their expression [35,36]. Moreover, regulating TEs by small interfering RNAs (siRNAs) is believed to be an epigenetic mechanism for balancing the expression and repression of stress-responsive genes of plant survival [28,37]. Still, this aspect of Al tolerance in cereals is poorly understood, even though it may significantly affect the trait. Studies in wheat, maize, and rice suggest that the expression of aluminum-related genes is regulated by DNA methylation of transposable elements (TEs) [29,38,39]. DNA methylation changes due to Al stress may, at least, modulate the expression profiles of the genes coding organic acids transporters, as was demonstrated in barley for the HvAACT1 gene responsible for citrate secretion [35]. A multiretrotransposon-like (MRL) sequence insertion detected in the upstream genomic region of the HvAACT1 gene significantly enhanced its expression in the root tips of Al-tolerant accessions. Furthermore, in accessions with low levels of HvAACT1 expression, this MRL insertion was present but highly methylated [35]. Similarly, Al stress promotes demethylation in the coding region of wild tobacco plants’ glycerophosphodiesterase-like protein gene (NtGPDL), enhancing expression [40]. Another study showed that overexpression of the S-adenosylmethionine (SAM) in AvSAMS1-transformed Arabidopsis plants impacts changes in DNA and histone H3 methylation after exposure to Al [41]. In rice exposed to Al stress, there were 26 genes that, at the transcriptomic level, differentiated tolerant and non-tolerant plants. The genes appeared to be 5 to 10 times more methylated in a tolerant cultivar than in a susceptible one [29].

Interestingly, transgenerational inheritance of epigenetic modifications has been observed in plants, suggesting that epigenome-targeted breeding strategies might enhance crop resistance to abiotic stresses [29,42,43]. Furthermore, epigenetic changes may not necessarily affect genes encoding the trait. They may impact multiple genes, influencing various cellular processes, such as ion transport, reactive oxygen species (ROS) detoxification, or hormone signaling [29,44]. Thus, further studies are necessary to gain a better understanding of how plants respond to abiotic stresses and how epigenetic regulation can be harnessed to improve crop tolerance [30,42].

Several molecular methods are available for studying epigenetic aspects of DNA methylation changes due to abiotic stresses. Two of them, namely Methylation Sensitive Amplification Polymorphism (MSAP) [45] and methylation Amplified Fragment Length Polymorphism (metAFLP) [46], are based on the AFLP technique [47] and utilize restriction enzymes with different specificity to DNA methylation [45]. The methods may utilize different approaches to quantify changes. MSAP, in its initial form, uses the so-called transitions for quantification, whereas semi-quantitative solutions may quantify methylation changes based on quantifying individual methylation changes affecting restriction sites. In its original form, the methods are dedicated to DNA methylation changes. The exception is the metAFLP approach, which may quantify sequence and DNA methylation alterations in all symmetric and asymmetric contexts. Unfortunately, the methods miss information on marker sequence. The disadvantage could be overcome by utilizing the DArTseqMet approach [48], which uses the same enzymes as MSAP and additionally delivers sequence data that could be processed in further analyses when combined with the semi-quantitative MSAP approach. Quantitative information regarding symmetric sequence contexts could be incorporated into data analysis.

This study aims to verify whether distinct ways of quantifying markers into variation types using the MSAP approach combined with DArTseq markers deliver comparable information on DNA methylation changes in plant materials affected by Al stress to illustrate putative distinctions or similarities of the systems. Furthermore, we wish to identify which types of DNA methylation changes related to symmetric DNA methylation contexts mainly affect Al tolerance and whether such markers are located close to the Al-tolerant genes or not.

## 2. Results

### 2.1. Plant’s Response to Aluminum Stress

Based on the Al physiological test, eight and seven lines were classified as tolerant (T) or non-tolerant (NT). The average root regrowth ranged from 0.9 to 2.9 cm for T lines, and trace or no regrowth was observed in the case of NT lines (Table 1). According to the physiological Al test results, there were seven NT and eight T lines. Furthermore, seven lines were classified as spring (S) and eight as winter (W). The root tips (1) and leaves (2) tissues were used in further analyses.

### 2.2. MSAP Analysis

The raw number of DArTseqMet markers equaled 17,177. Based on the frequency of the marker presence among plants representing the line identified in SbfI/HpaII and SbfI/MspI digests, the presence or absence of a marker is indicated. As an outcome, four-digit codes were generated and used for DNA methylation changes affecting HpaII-MspI site quantification, following the reasoning illustrated in Supplementary Table S1. Depending on the quantification model, fluctuations in quantitative characteristics were evidenced (Table 2). 

In general, the widely used model (Common) [45] resulted in lower values of DNA demethylation (DM), de novo DNA methylation (DNM), DNA methylation status preservation (MP), and total methylation (M) of the HpaII-MseI restriction sites compared to the data gained in the General model of its basic variant. However, non-methylation preservation (NMP) and non-methylation (NM) contained higher values in the Common than the General basic model, independent of whether root tips or leaf tissues were analyzed. As an extended variant of the General model is part of a basic one but delivers information on DNA de novo methylation and DNA demethylation affecting CG and CHG sequence contexts of the restriction sites, the respective values were the lowest compared to other quantitative data. The highest standard deviation values affected DNA methylation quantitative characteristics of the Common model, whereas the lowest was typical for the extended variant of the General one (Table 3).

Significant Pearson’s correlation coefficients (Supplementary Table S2) were evaluated for DNA demethylation (DM) characteristics evaluated in all models. They were negatively correlated with NMP and positively correlated with MP. The correlation between NMP and MP was lower (from −0.503 to −0.803) than between DM and NMPs (from −0.783 to −0.937). Furthermore, a high correlation level was evaluated for all DNM (Common and basic variants of the General model) and DN affecting CG and CHG sequence contexts (from 0.892 to 0.962). DN and DNM were negatively correlated with NM, whereas NMs were positively correlated in each model.

### 2.3. ANOVA Analysis

ANOVA indicated some differences between the models regarding the extent to which tolerant/non-tolerant and winter/spring lines, as well as root tips/leaf tissues, explain quantitative variables describing different aspects of DNA methylation due to Al treatment. In all models tested, the ANOVA was significant (α ≤ 0.05). In the Common model, four main effects due to the winter–spring trait of the lines were detected, but only those associated with M and NM were significant. Similarly, in the General model (basic variant), four main effects related to winter–spring lines in the cases of DNM, MP, M, and NM were also revealed. Finally, in the extended variant of the General model, the main effects were also due to winter–spring lines in the cases of de novo DNA methylation of both symmetric sequence contexts.

Furthermore, the quantitative characteristics were explained by either two-way or three-way interactions. Two-way interactions were preferentially represented by tolerance and tissue and rarely by tolerance and winter–spring lines. The highest value of explained variance was 0.753 (M and NM variables of the basic general model), whereas the lowest was 0.281 (DNM-Common model) (Table 4).

### 2.4. Elastic Net Regression Analyses

The employment of elastic net regression showed that in the Common model, DNM and DM were insignificant in explaining Al tolerance in contrast to non-tolerance, spring in contrast to winter lines, and in describing the difference between root and leaf tissue (Table 5). However, NMP and M described tolerant and non-tolerant materials, with the prevailing positive effect of M in non-tolerant materials and the negative effect of NMP. When the basic variant of the general model data was employed in the analysis, DNM proved significant in describing tolerant and non-tolerant lines. Furthermore, the negative effect of NMP and M’s positive effect in non-tolerant lines were evaluated. The extended variant of the general model showed that DM-CG, DN-CHG, and DN-CHG positively affect non-tolerant lines. Thus, demethylation and de novo methylation negatively impact Al tolerance. The observed effects are mostly related to the CG sequence contexts.

### 2.5. Distribution of Markers Assigned to DN-CG, DN-CHG, and DM-CG Regarding Tolerance

**Figure 1:**
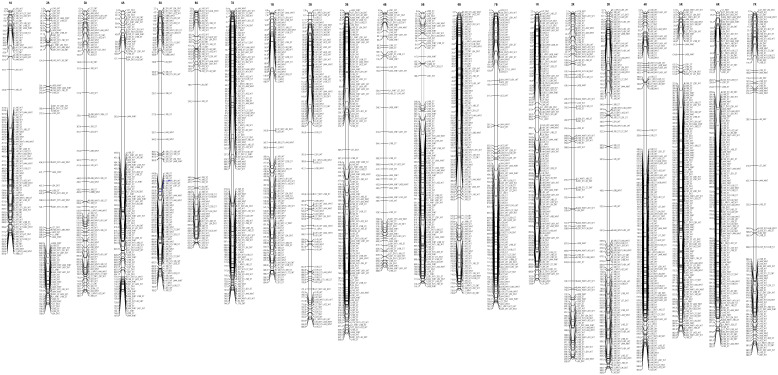
Representation of the genetic map of triticale illustrating the location of the DN-CG-assigned DArTseqMet markers univocally classified to the DN events within CG sequence contexts (1101) in root tips (<0.5 cm). Scale on the map: 1.0 = 1 mln bp. W and S indicate winter and spring forms, and NT and T reflect non-tolerant and tolerant lines.

**Figure 2:**
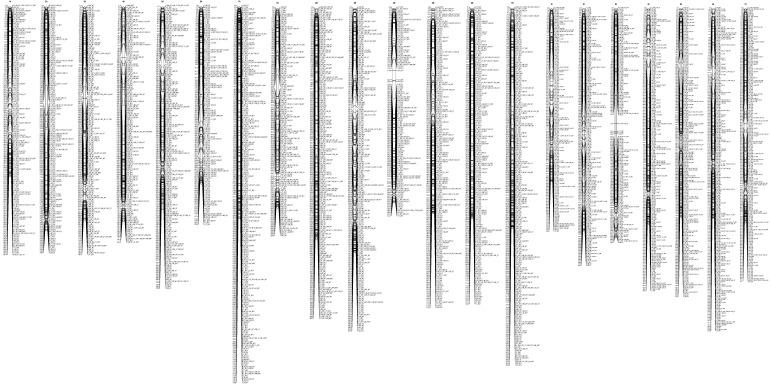
Representation of the genetic map of triticale illustrating the location of the DM-CG - assigned DArTseqMet markers univocally classified to the DM events within CG sequence contexts (0111) in root tips (<0.5 cm). Scale on the map: 1.0 = 1 mln bp. W and S indicate winter and spring forms, and NT and T reflect non-tolerant and tolerant lines.

**Figure 3:**
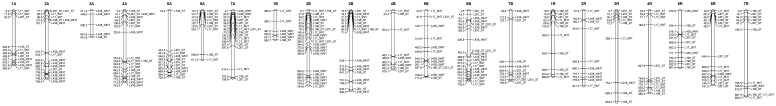
Representation of the genetic map of triticale illustrating the location of the DN-CG-assigned DArTseqMet markers univocally classified to the DN events within CG sequence contexts (1101) in leaves. Scale on the map: 1.0 = 1 mln bp. W and S indicate winter and spring forms, and NT and T reflect non-tolerant and tolerant lines.

**Figure 4:**
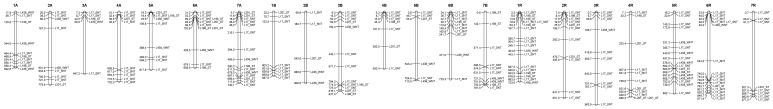
Representation of the genetic map of triticale illustrating the location of the DM-CG-assigned DArTseqMet markers univocally classified to the DM events within CG sequence contexts (0111) in leaves. Scale on the map: 1.0 = 1 mln bp. W and S indicate winter and spring forms, and NT and T reflect non-tolerant and tolerant lines.

**Figure 5:**
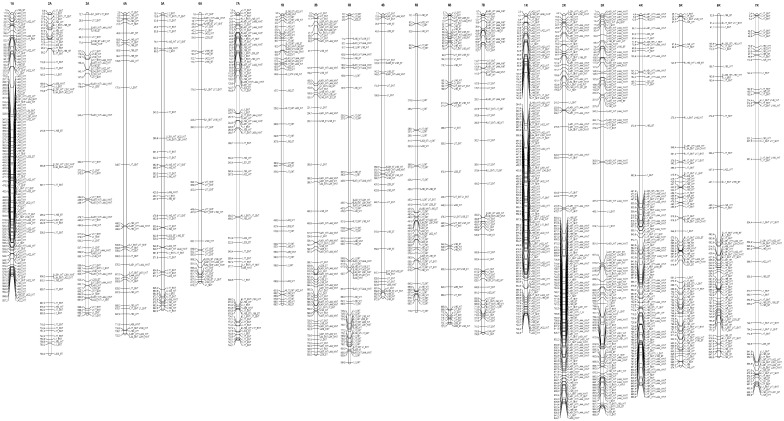
Representation of the genetic map of triticale illustrating the location of the DN-CHG-assigned DArTseqMet markers univocally classified to the DM events within CG sequence contexts (1110) in root tips (<0.5 cm). Scale on the map: 1.0 = 1 mln bp. W and S indicate winter and spring forms, and NT and T reflect non-tolerant and tolerant lines.

**Figure 6:**
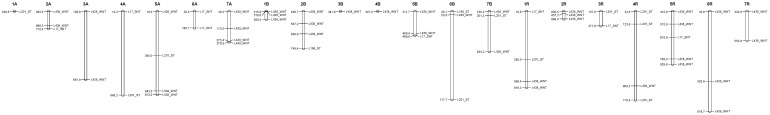
Representation of genetic map of triticale illustrating the location of the DN-CHG-assigned DArTseqMet markers univocally classified to the DM events within CG sequence contexts (1110) in leaves. Scale on the map: 1.0 = 1 mln bp. W and S indicate winter and spring forms, and NT and T reflect non-tolerant and tolerant lines.

The DArTseqMet markers assigned to the DNA de novo events and DNA demethylation affecting CG sequence contexts and mapped to the triticale chromosomes (based on reference maps) were not evenly distributed along chromosomes. Furthermore, they were differently represented in root tips (Figure 1 and Figure 2) and leaves (Figure 3 and Figure 4). Comparable distribution was observed in the case of DN-CHG markers (Figure 5 and Figure 6). In all cases, the number of markers identified in root tips was much higher than the number of markers reflecting leaves, indicating that the Al stress is transmitted to leaves to a limited extent. The markers related to root tips are preferentially mapped to telomeric regions, sometimes forming tightly linked blocks, establishing putative hotspots of epigenetic changes. The same was observed for markers identified in leaves. 

## 3. Discussion

### 3.1. Uniformity of Plant Materials

The current study is based on at least 40 individual plants representing each line analyzed to ensure that a sufficient amount of root tissue was collected for the analysis. There were three biological repetitions of such experiments. We have chosen root tips as the tissue directly in contact with the Al-containing medium, whereas leaves were collected because stress could be transmitted to other tissues [49,50]. However, the extent of such transmission is not known. The lines were selected for at least seven generations via selfing each plant as a starting point for the next generation. Thus, all further analyses were expected to be based on genetically uniform materials. Furthermore, as all Al-treated lines were subjected to the same stress level (the experiment that allowed tissue collection), it was anticipated that the seventh (and subsequent) generations would have identical genotypes. Phenotypic analysis of the lines based on the Al test confirmed that tolerant and non-tolerant materials had stable phenotypes, as indicated by the root regrowth data.

### 3.2. Characteristic of DArTseqMet Approach for DNA Methylation Study

Currently, several approaches that allow quantification of DNA methylation changes based on the MSAP approach [51,52,53] are available. Depending on how the molecular profiles are interpreted, the approaches can be classified as Common and General (including basic and extended variants) (Supplementary Table S1). The Common approach assumes the most probable explanation, omitting other alternatives. The transition resulting in such an interpretation is classified into a specific type and counted. Contrarily, the general approach tries to interpret differences between profiles based on putative changes affecting the site’s cytosines. It can also deliver information on changes affecting symmetric sequence contexts regarding DNA methylation changes, giving information on epigenetic aspects of Al stress. Unfortunately, the limitation of the approaches is the lack of information regarding DNA marker sequences if the AFLP markers are used. Therefore, an adequate marker system allowing marker classification to varying DNA methylation characteristics and their quantification is needed. Moreover, preferences should be given to the marker system that allows mapping utilizing its information on chromosomal location.

The DArTseqMet approach is based on the HpaII/MspI isoschizomer and delivers marker sequence information. If combined with MSAP or semi-quantitative MSAP, the changes could be quantified and normalized. Furthermore, the analysis allows the evaluation of the (epi) genetic background reflecting the four-digit codes regarding CG and CHG sequence contexts. Thus, detailed information on subtle DNA methylation changes related to the methylation alterations transferred during DNA replication and those reflecting some epigenetic aspects could be extracted.

The general approach, which utilizes many DArTseqMet markers, delivers reliable and extensive information on epigenetic changes affecting DNA methylation patterns. It was demonstrated that differences were present using varying MASP characteristics evaluated in the Common or General models. For example, higher DM, DNM, MP, and M values were evaluated compared to the Common model, whereas NMP and NM values were higher in the Common model. The differences are due to how the two models quantify respective differences, with the Common approach underestimating some changes. The values of DM and DN related to the CG and CHG contexts of the extended variant were lower than those of DN and DNM evaluated in the other cases, which is not unexpected, as only a tiny fraction of changes were considered (due to the low representation of changes related to CHH sequence context). It is also unsurprising that the data evaluated based on all methods exhibit varying correlations (Supplementary Table S2), with only a few cases above 0.8. Still, correlated data indicate that models reflect the same phenomenon with varying approximations.

### 3.3. Epigenetic Alterations in Triticale Under Al Stress

As could be seen, all quantitative characteristics analyzed in the General model were significant, whereas in the Common one, the model encompassing DNM% was insignificant. Furthermore, in the Common and General models, there were some differences in the presence of the ANOVA’s main effects (Table 4); however, in nearly all cases, the main effect was due to the winter/spring lines. Regarding the extended variant of the general model, all characteristics resulted in significant ANOVA, with only DN-CG% and DN-CHG% having the main effect in winter/spring materials. In the case of all statistical models, at least two-way interactions, primarily due to tolerance and tissue, were found. Thus, although analyses based on the Common and General models reflect the same phenomenon, they differ in detail, suggesting that it is crucial to apply the best one that fits experimental requirements and scientific demands. Apparently, however, ANOVA supports the notion regarding epigenetic alterations in triticale DNA under Al stress.

As indicated earlier, phenotypic stability reflecting Al tolerance was evaluated for the materials tested. Such a result might reflect genotypic stability due to the Al-tolerant gene we mapped earlier to the 7R chromosome, which explains approximately 36% of phenotypic variance [26]. Besides, in triticale, the presence of the QTLs responsible for the trait on 3R, 4R, 5R, and 6R indicates that the other genes may influence the trait [54]. Moreover, the heritability coefficient in the broad and narrow sense was determined for twenty triticale genotypes presented at the International Maize and Wheat Improvement Centre (CIMMYT), and they varied from 63.77 to 91.80 and from 74.46 to 94.56, respectively [55]. Such results indicate that simple inheritance is governed by a dominant gene and the presence of additional factors that influence trait expression. Thus, an alternative explanation is that during subsequent inbreeding steps, epigenetic aspects of Al tolerance were also stabilized. The notion is supported by studies indicating the role of epigenetics expressed at the DNA methylation level regarding rice, wheat, barley, and maize and showing fluctuations in methylation patterns within differentially expressed genes [29] or transposons [35,38,39] due to Al treatment.

The question is whether or not DNA methylation changes regarding metal stress are related to the respective tolerant gene coding sequences. Based on available data, DNA hypomethylation was observed at the promoters of the Heavy Metal ATPase 2 (TaHMA2) and ATP-Binding Cassette (TaABCC2/3/4) metal detoxification transporters in the resistant wheat genotype Pirsabak 2004 compared with the control in response to Pb, Cd, and Zn [56]. Besides, regulation by DNA methylation under heavy metal stress is not restricted to the promoter regions of tolerant genes but is also observed in their coding regions and TEs. TEs have been implicated in Al stress responses in barley [35]. In the Al-tolerant genotype of barley, the multiretrotransposon-like (MRL) insertion and the expression of the HvAACT1 gene responsible for coding citrate transporters are due to demethylation processes. Additionally, transposon insertions close to genes have been proposed as a source of epialleles and a mechanism affecting specific genes’ transcriptional regulation [35]. 

Interestingly, epigenetic changes are also present in the case of other stresses and appear in the gene body or its vicinity, affecting the gene’s transcription. A set of 36 randomly selected genes that underwent DNA methylation changes under drought in barley showed a high modulation at the transcriptome level [44]. For example, the expression of MLOC_61723 decreased more than 200 times under water deficiency and returned to the basal level after the rewatering phase. Another example comes from in vitro tissue cultures. It was shown that even poorly androgenic DH rye lines may become androgenic if they pass several anther culture cycles of plant regeneration [57]. However, there is no information on whether methylation changes affect specific genes or are dispersed along species chromosomes.

### 3.4. Distribution of the Epigenetic Markers Linked to Al Tolerance on the Triticale Map

It is not apparent whether DNA methylation changes related to Al stress impact the whole genome or affect regions where respective Al-tolerant genes were mapped, suggesting that either the whole genome is involved in stress response or that the genome is tuned at the regions of trait coding genes linking epigenetic markers and genetic traits [14,35]. In the former case, epigenetic changes might be less specific and reflect a minute response to the stress. Thus, epigenetic differences between control lines and Al-treated counterparts could be evaluated. We have also suspected that epigenetic mechanisms preferentially act on a density level [52], meaning that DNA methylation may not always affect the same cytosines, but depending on the density of mapped markers, the two alternatives could be differentiated. Alternatively, genetic and epigenetic factors work cooperatively to support Al tolerance in triticale. If the latter hypothesis is valid, markers differentiating control and Al-treated lines should be mapped near Al-tolerant QTLs. Those DArTseqMet markers univocally assigned to the given type of event and found differentiating control and treated lines based on ANOVA were mapped based on their known chromosomal location [58] to distinguish exclusively between the alternatives discussed. Thus, only DNM-CG-, DNM-CHG-, and DM-CG-assigned markers were used for map construction. As can be seen, markers are distributed along all chromosomes, with some preferences towards telomeric regions. The distribution is comparable to markers detected for root tips and leaves. Furthermore, no evident increased marker density is found near known Al-tolerant QTLs related to the Al stress response in triticale [14,54]. Thus, DNA methylation changes reflect the global tuning of the triticale genome to Al stress rather than being associated with the functioning of Al-tolerant genes. Still, the presented data do not necessarily exclude directed effects, as the observed dispersion of markers may reflect the tuning of specific biochemical pathways or gene expression patterns supporting tolerance. 

However, an alternative explanation regarding Al tolerance is also possible. It is well-known that aluminum stress may alleviate Reactive Oxygen Species (ROS) production. Studies on rice (*Oryza sativa*) revealed that Al^3+^ ions generate H_2_O_2_ or O_2_ particles, which leads to DNA damage, lipid peroxidation, or even cell death in root cells [59,60], disturbing the proper functioning of organisms. However, plants’ antioxidant system is capable of eliminating ROS. Several enzymes like peroxidase (POD), catalase (CAT), ascorbate peroxidase (APX), superoxide dismutase (SOD), glutathione reductase (GR), and glutathione peroxidase (GPX) take part in the recovery of ROS-mediated damages. The same function is also assigned to glutathione (GSH), ascorbate, and proline [61]. Additionally, Al ions, which are not bound in the cell wall and cell membrane, can enter the cell and directly cause DNA damage by binding to the phosphoric acid residues in DNA. Recent studies demonstrate that aluminum leads to the induction of the DNA Damage Response (DDR) pathway, which is highly specialized in detecting DNA damage [62,63]. 

Further analysis of marker sequences and identification of biochemical, transcriptional, and other cellular processes are needed to confirm any hypothesis.

### 3.5. Tissue-Specific Methylation Level in Triticale Under Al Stress

An interesting aspect of this study is that it reflects somatic memory. An analysis of maps constructed on DArTseqMet markers shows that the density of root tip-based maps is much higher than leaf-based maps. It is what we expect if a stress signal is transmitted to other tissues. Whether the markers identified for leaves reflect random effects or may represent the most significant genomic regions linked to Al tolerance needs further investigation. Contrary to our results, Liu et al. [50] showed that more DNA methylations occurred in leaves than in roots of sunflowers under salt and alkali stress. However, roots carried more CG or CHG variations under each stress, indicating that roots play the central part of a sunflower plant’s response to various stresses [50]. Drought stress mainly induces demethylation events in leaves, whereas novel methylations are more abundant in roots [44]. But, after the rewatering phase, more new methylations were induced in leaves, whereas there were more demethylations in roots. Such organ-specific methylome changes might regulate the drought resistance in barley. Other researchers suggested that distinct genes regulated tissue-specific biological functions and eventually involved differential DNA methylations [49,64]. Further analysis of sequence data may be helpful in a deeper understanding of the phenomenon in triticale.

### 3.6. Elastic Net Regression Analyses

An essential aspect of this study is the employment of elastic net regression analysis, a valuable method used to detect the influence of different variables in explaining a trait. As expected, somewhat different variables’ effects were evaluated depending on whether the Common or general (basic variant) model characteristics were used to explain differences between non-tolerant and tolerant or spring and winter lines. When the General (basic variant) approach data were analyzed to explain T/NT, DNM, and M, negative and positive effects on Al-non-tolerant lines were evaluated. Except for DNM, the Common and basic general models were congruent regarding effects and their signs. However, the extended model revealed that DNM was related to the CG context, and DN-CG and DN-CHG affected T-NT. Interestingly, the effect of DM-CG was close to that of DN-CG, whereas DN-CHG had the lowest effect. This indicates that most DNA methylation changes revealed in the analysis were due to the context of CG. 

## 4. Materials and Methods

### 4.1. Plant Materials

Eight spring and seven winter triticale lines represented by at least 40 plants were subjected to the Al test. There were three biological repeats of each of the tests. The lines were highly homozygotic (F7–F10 generations) and screened for Al tolerance annually. Breeders kindly delivered plant materials from Strzelce Plant Breeders Ltd., Experimental Station Małyszyn, 66-400 Gorzów Wielkopolski, Poland.

### 4.2. Physiological Test

Triticale seeds were soaked successively in alcohol (1 min), 20% bleach solution (Domestos, Unilever, Polska), and distilled water (5, 10, and 15 min). The moist seeds were germinated on a paper filter in a petri dish for 24 h at 20 °C in the dark. Germinated seeds were transferred to nets floating on a basic medium (2.0 mM CaCl_2_, 3.25 mM KNO_3_, 1.25 mM MgCl_2_, 0.5 mM (NH_4_)2SO_4,_ and 0.2 mM NH_4_NO_3_, pH 4.5) in a plastic tray [65]. The seedlings were maintained under a controlled environment (temperature 25 °C, photoperiod 12/12 h day/night, light intensity 40 W/m^2^) in a growth cabinet (POL-EKO-APARATURA, ST500 B40 FOT10). After three days, the seedlings were transferred onto the same medium containing aluminum ions (16 ppm; AlCl_3_ × 6H_2_O) for 24 h. The plants were washed in water and placed in the basic medium for 48 h. Al tolerance is based on the ability of seedlings to continue root growth and was therefore evaluated by measuring root regrowth. The root tips 0.3–0.5 cm in length (1), and leaves (2) from 7-day-old seedlings, both exposed and non-exposed to Al^3+^ ions, were excised and used for DNA isolation.

### 4.3. DNA Isolation

Total genomic DNA was isolated from bulked samples representing all Al-tested plants, reflecting given root tips and leaves as separate samples of a given line, using the Plant DNeasy MiniKit 250 (Qiagen, Hilden, Germany) following the manufacturer’s instructions. DNA quantity was measured spectrophotometrically (NanoDrop ND-1000, Wilmington, DE, USA), and its integrity and purity were tested on a 1.2% agarose gel in TBE buffer. The isolations from each biological repeat were conducted separately and subjected to further analysis independently.

### 4.4. Methylation Sensitive DArT Sequencing—DArTseqMet

DNA markers were generated at Diversity Arrays Technology Pty Ltd., Canberra, Australia. DArTseq is an efficient genotyping-by-sequencing technology based on next-generation sequencing (NGS), allowing the discovery of whole-genome sequencing markers [58]. For methylation analysis, the Methylation Sensitive Amplification Polymorphism (MSAP) method, which uses isoschizomers with different specificities for DNA methylation at the restriction site, was adapted to the DArTseq technology [48]. Two parallel libraries were prepared per sample using a DNA double-digestion restriction-based protocol, combining the enzymes SbfI and MspI into the first and SbfI and HpaII into the second platform. DNA samples were processed in digestion/ligation reactions as Kilian et al. [58] described, but with adaptors compatible with restriction enzyme (RE) in brackets. Then, SbfI/MspI and SbfI/HpaII fragments were effectively amplified through PCR. Amplification products were single-end sequenced in an Illumina HiSeq 2500. The raw DArTseqMet marker did not contain missing data.

### 4.5. DArTseqMet Markers Interpretation and Quantification Models

It is assumed that the presence of a given marker sequence means that the given marker has the respective restriction site that the given enzyme could digest and that its putative methylation does not affect digestion. Furthermore, the presence of a given digested DArTseqMet marker in any digests (control/Al-treated) means that the sequence is available in any case, and that it is missing should be interpreted in terms of cytosine methylation that blocks digestion. Following that reasoning, the presence of the marker is coded as one (1), whereas its absence is coded as zero (0). As control is compared to its Al-treated counterpart, and there are two types of digests, each marker is reflected by a four-digit code reflecting distinct restriction site methylation status that could be distinctly evaluated employing varying quantitative models of analysis or DArTseqMet markers based on the MSAP or semiquantitative MSAP approaches. Under such conditions, mutations are not considered a source of variation.

Thus, two quantitative models could be distinguished. One is based on the original MSAP procedure (the Common model), and the other is based on the semi-quantitative MSAP variant. The latter may be employed using essential quantitative characteristics, and if needed, more details related to sequence contexts could be extracted (extended variant). The main differences between the models are given below (Supplementary Table S1):

1. The Common model is based on the prevailing methylation/non-methylation state/status background explanations for the given transition types, which are used for methylation quantification purposes.
2. The General model:
  - The Basic variant assumes that all possible explanations regarding the methylation background that stands behind the MSAP profiles are used for calculations following the approach presented earlier [52];
  - The Extended variant is based on the same assumptions as the Basic one, but a detailed analysis of restriction site methylation status is conducted to address them in sequence context.

### 4.6. Statistics

Descriptive statistics, Pearson’s correlation coefficients, 3-way ANOVA, and Elastic net regression analyses were performed using XlStat ver. 2024.3.0 software [66]. ANOVA was employed to examine how categorical variables (Al tolerance, winter–spring lines, and plant tissues) affected the quantitative variables evaluated based on the Common and General (basic and extended variants) models of quantifying DNA methylation. The Elastic net regression analysis with an alpha parameter set to one included a single categorical variable as a dependent variable, including all quantitative variables of the Common and general models as explanatory variables.

### 4.7. Map Construction

The MSAP markers were mapped to the triticale chromosomes based on their known positions delivered by Diversity Arrays Technology Pty Ltd., Canberra, Australia. Only markers showing the same methylation pattern (for example, ‘0111’ for CG demethylation and ‘1101’ for CG de novo methylation) were mapped, at least for two of the three experiment repetitions for the given triticale line. MapChart ver. 2.3 software was used to draw the genetic linkage maps [67].

## 5. Conclusions

The presented study demonstrates that utilizing different methods to quantify DNA methylation changes induced by Al stress, although congruent, differ in details, and the results evaluated based on them should be compared critically. The Common basic general approach is sufficient if general information is needed. However, when a deeper insight into the phenomenon is required, it is recommended to use an extended general approach. Using the General (extended variant) approach in combination with ANOVA, the differences between Al-treated and control lines regarding DN-CG, DM-CG, and DN-CHG were detected. Analysis of the maps constructed based on those markers showed that they were located in telomeric regions without grouping in the vicinity of the Al-tolerant QTLs, suggesting that Al-treatment induces minute changes in the genome. Still, other explanations are not excluded. Moreover, genetic maps constructed based on root tips and leaf tissues differed in density but reflected the comparable patterns of their distribution, supporting the hypothesis that Al stress could be transmitted to other plant tissues due to somatic memory. Further analyses of marker sequences are needed to ensure or disclose whether epigenetic changes due to Al stress are random or whether they activate/deactivate processes supporting genetic factors of tolerance.

## Figures and Tables

**Table 1:** The aluminum test results illustrating the average root regrowth (RG) in cm measured after 24 h of Al treatment followed by 48 h of growing under unstressed conditions.

Line	L195	L198	L201	L203	L1	L17	L27	L34	L145	L190	L422	L451	L291	L438	L444
Cl *	S/T	S/T	S/T	S/T	S/NT	S/NT	S/NT	S/NT	W/T	W/T	W/T	W/T	W/NT	W/NT	W/NT
RG	1.8 ± 0.4	2.9 ± 0.4	2.8 ± 0.5	0.9 ± 0.2	0.1 ± 0.1	0.0 ± 0.0	0.0 ± 0.0	0.0 ± 0.0	2.2 ± 0.3	1.7 ± 0.2	2.1 ± 0.3	1.9 ± 0.4	0.1 ± 0.1	0.0 ± 0.0	0.0 ± 0.0

* Cl—classification; RG—root regrowth; S and W state for spring and winter lines; T and NT reflect tolerant and non-tolerant lines.

**Table 2:** The MSAP-based quantitative characteristics utilizing DArTseqMet markers reflecting analyzed plant materials and tissues.

Plant Materials			Common Model						General Model									
									Basic						Extended			
Tolerance	S/W	Plant Part	DM (%)	DNM (%)	MP (%)	NMP (%)	M (%)	NM (%)	DM (%)	DNM (%)	MP (%)	NMP (%)	M (%)	NM (%)	DN-CHG (%)	DN-CG (%)	DM-CHG (%)	DM-CG (%)
T	S	R	4.25	3.26	33.38	59.92	36.34	63.66	9.79	9.11	36.27	44.82	45.38	54.62	0.86	8.22	1.32	8.48
T	S	R	4.64	2.74	33.69	60.09	36.01	63.99	9.56	8.79	37.24	44.40	46.03	53.97	0.55	8.19	0.87	8.69
T	S	R	2.14	3.89	33.47	62.46	36.64	63.36	8.53	9.43	36.88	45.16	46.31	53.69	0.88	8.45	0.60	7.93
T	S	R	1.82	5.37	35.00	60.24	39.41	60.59	8.53	10.56	37.82	43.08	48.38	51.62	1.41	9.01	0.44	8.10
NT	S	R	2.55	7.65	34.29	59.21	40.45	59.55	8.88	11.70	36.84	42.59	48.53	51.47	2.03	9.55	0.65	8.23
NT	S	R	1.78	9.49	35.74	57.15	43.40	56.60	8.52	12.57	37.25	41.66	49.82	50.18	2.13	10.31	0.45	8.07
NT	S	R	6.65	4.63	38.86	51.64	42.73	57.27	12.42	10.37	40.45	36.76	50.82	49.18	1.28	9.01	2.68	9.73
NT	S	R	7.04	3.92	38.94	51.06	42.45	57.55	12.14	9.95	40.33	37.58	50.28	49.72	1.04	8.78	2.40	9.74
T	W	R	1.74	4.23	35.32	60.81	38.73	61.27	8.50	9.89	37.80	43.81	47.69	52.31	1.11	8.70	0.32	8.18
T	W	R	2.45	5.73	35.09	59.59	39.69	60.31	8.73	10.63	37.58	43.06	48.21	51.79	1.32	9.21	0.58	8.15
T	W	R	2.19	6.66	35.02	57.17	41.25	58.75	8.78	11.71	38.25	41.26	49.96	50.04	2.14	9.40	0.51	8.27
T	W	R	1.52	4.82	36.84	60.01	40.38	59.62	8.52	10.03	39.23	42.21	49.27	50.73	0.60	9.40	0.31	8.21
NT	W	R	12.92	9.34	35.26	44.04	43.92	56.08	14.93	12.40	38.46	34.22	50.85	49.15	2.18	10.09	4.26	10.67
NT	W	R	10.06	4.97	34.23	49.52	39.69	60.31	12.59	11.02	40.53	35.86	51.55	48.45	1.62	9.31	3.08	9.51
NT	W	R	6.75	4.41	39.27	51.21	42.97	57.03	12.40	10.20	40.65	36.75	50.85	49.15	1.09	9.02	2.65	9.75
NT	S	L	3.19	3.48	35.75	58.06	39.04	60.96	9.23	9.64	38.94	42.18	48.58	51.42	0.84	8.72	0.69	8.55
T	S	L	3.11	3.85	33.01	61.34	36.39	63.61	8.87	9.35	36.82	44.96	46.16	53.84	0.77	8.51	0.76	8.11
T	S	L	8.16	2.98	33.73	56.81	36.10	63.90	12.18	8.55	37.04	42.24	45.58	54.42	0.29	8.22	2.94	9.24
NT	W	L	3.91	5.85	35.88	54.76	41.61	58.39	9.79	11.30	38.95	39.96	50.25	49.75	1.84	9.37	0.88	8.91

Lines are encoded as NT (Al non-tolerant) and T (Al tolerant). Spring lines are indicated as S, whereas winter as W. Plant parts reflect root, where R is the root tip regrowth, and leaf tissue, indicated by L. DM—DNA demethylation, DNM—DNA de novo methylation, MP—methylation preservation (sites that were methylated in control and treated lines); NMP—non-methylated sites (as MP but regarding non-methylated sites); M—MP plus DNM; NM—NMP and DM; CG and CHG indicate two types of symmetric sequence contexts. The General model assumes that the whole genetic background explaining the MSAP four-digit code was used to quantify DN, DNM, M, MP, NM, and NMP characteristics employed for calculations. The sequence contexts model is similar to the General model. However, DM and DNM characteristics were evaluated in symmetric sequence contexts. Finally, the Common model is similar to the General one except that the most abundant explanation of the four-digit MSAP profile was used for calculation.

**Table 3:** The arrangement of basic statistics concerning MSAP quantitative characteristics.

Model		Variation Type (%)	Minimum	Maximum	Mean	SD
Common		DM	1.52	12.92	4.57	3.21
		DNM	2.74	9.49	5.12	1.96
		MP	33.01	39.27	35.41	1.89
		NMP	44.04	62.46	56.58	4.93
		M	36.01	43.92	39.85	2.64
		NM	56.08	63.99	60.15	2.64
General	Basic variant	DM	8.50	14.93	10.15	1.96
		DNM	8.55	12.57	10.38	1.16
		MP	36.27	40.65	38.28	1.42
		NMP	34.22	45.16	41.19	3.36
		M	45.38	51.55	48.66	1.99
		NM	48.45	54.62	51.34	1.99
	Extended variant	DN-CHG	0.29	2.18	1.26	0.56
		DN-CG	8.19	10.31	9.06	0.59
		DM-CHG	0.31	4.26	1.40	1.20
		DM-CG	7.93	10.67	8.76	0.78

**Table 4:** The arrangement of ANOVA results with quantitative variables of each model treated as dependent variables and qualitative ones as explanatory variables.

Model	Dependent Variable	ANOVA Statistics Description	Main Effects/Interactions	Statistics					
				*MSE*	*MS*	*F* *(2,16)*	*p*	*R* ^2^	*R* ^2^ *_adj_*
Common	DM%	Model		4.95	53.08	10.73	0.001	0.573	0.519
		interactions	tolerance * tissue		27.66	5.59	0.008		
			tolerance * W-S * tissue		50.08	10.12	0.006		
	DNM%	Model		2.76	12.52	4.53	0.028	0.361	0.281
		interactions	W-S		7.81	2.83	0.110		
			tolerance * W-S * tissue		19.53	7.07	0.017		
	MP%	Model		2.3	13.72	5.96	0.011	0.427	0.355
		Main effects	W-S		1.94	0.84	0.370		
		interactions	tolerance * W-S		24.58	10.68	0.005		
	NMP%	Model		8.05	153.97	19.12	0.0001	0.700	0.668
		interactions	tolerance * tissue		94.65	11.75	0.0002		
			tolerance * W-S * tissue		72.61	9.02	0.008		
	M%	Model		2.45	43.00	17.55	0.0001	0.686	0.647
		Main effects	W-S		22.66	9.25	0.008		
		interactions	tolerance * W-S * tissue		66.84	27.28	0.00008		
	NM%	Model		2.45	43.00	17.54	0.00009	0.686	0.647
		Main effects	W-S		22.66	9.25	0.008		
		interactions	tolerance * W-S * tissue		66.85	27.28	0.00008		
General (basic variant)	DM%	Model		1.81	20.03	11.08	0.001	0.581	0.528
		interactions	tolerance * tissue		11.71	6.48	0.004		
			tolerance * W-S * tissue		13.6	7.52	0.014		
	DNM%	Model		0.77	5.98	7.75	0.004	0.492	0.428
		interactions	W-S		4.96	6.42	0.022		
			tolerance * W-S * tissue		8.25	10.69	0.005		
	MP%	Model		1.13	9.10	8.07	0.004	0.5	0.44
		Main effect	W-S		5.1	4.53	0.049		
	NMP%	Model		3.26	75.7	23.22	0.00001	0.743	0.712
		interactions	tolerance * tissue		62.28	19.1	0.00005		
			W-S * tissue		32.88	10.08	0.006		
	M%	Model		0.98	27.85	28.49	0.0001	0.781	0.753
		Main effects	W-S		16.61	16.99	0.001		
	NM%	Model		0.98	27.85	28.49	0.0001	0.781	0.753
		Main effects	W-S		16.61	16.99	0.001		
General (extended)	DN-CHG%	Model		0.23	1.21	5.15	0.019	0.391	0.315
		Main effects	W-S		0.70	2.99	0.100		
		interactions	tolerance * W-S * tissue		1.71	7.3	0.016		
	DN-CG%	Model		0.19	1.69	8.91	0.003	0.526	0.468
		Main effects	W-S		1.19	6.28	0.023		
		interactions	tolerance * W-S * tissue		2.24	11.79	0.003		
	DM-CHG%	Model		0.69	7.43	10.79	0.001	0.574	0.521
		interactions	tolerance * tissue		9.4	13.65	0.002		
			tolerance * W-S * tissue		5.46	7.93	0.012		
	DM-CG%	Model		0.31	3.06	9.96	0.001	0.555	0.499
		interactions	tolerance * tissue		4.28	13.94	0.002		
			tolerance * W-S * tissue		1.83	5.97	0.026		

* interaction.

**Table 5:** The arrangement of the elastic net regression analyses, indicating variables and respective regression coefficients that were significant in explaining the classification of non-tolerant/tolerant lines and their winter–spring belonging.

Classification	DNA Methylation Quantitative Models								
	Common			General					
				Basic Variant			Extended Variant		
	T-NT	S-W	Root-Leaves	T-NT	S-W	Root-Leaves	T-NT	S-W	Root-Leaves
Optimal Lambda	0.0511	0.1931	0.1285	0.0578	0.0833	0.1244	0.0419	0.2083	0.1209
Intercept.	0.2028	−0.3184	−1.3218	5.1232	−19.9417	−1.3218	−38.316	−0.3185	−1.3217
DM	0	0	0	0	0	0			
DM-CHG							0	0	0
DM-CG							2.3539	0	0
DNM	0	0	0	0.2477	0	0			
DN-CHG							0.5351	0	0
DN-CG							1.8941	0	0
MP	0	0	0	0	0	0			
NMP	−0.2853	0	0	−0.4391	0	0			
M	0.4006	0	0	0.2128	0.4021	0			
NM	0	0	0	−1.6 × 10^−16^	−3.9 × 10^−16^	0			

## Data Availability

Data will be made available upon request.

## Supplementary Materials

The following supporting information can be downloaded at: https://www.mdpi.com/article/10.3390/ijms26114995/s1.
