# Supplementary material for: DNA Methylation Changes Reflect Aluminum Stress in Triticale and Epigenetic Control of the Trait
Source: Int J Mol Sci. 2025 May 22;26(11):4995. doi: 10.3390/ijms26114995 (PMC12154425; doi:10.3390/ijms26114995)
Supplement: Supplementary file 1 [file ijms-26-04995-s001.zip › ijms-3615265-supplementary/Table S/Table S1.pdf]

Table S1. The theoretical background used in the MSAP (common model), semi-quantitative MSAP (general model) with its basic and extended variants applied to DArTseqMet markers.

4-digit code /events	EVENTS' DESCRIPTION				Background (restriction site and transition symbol)			The MSAP-based (Common model) event classification	MSAP-based (Common model) transition-related events classification	The semi-quantitative MSAP-based (General (basic) model) - event classification	The semi- quantitative MSAP-based General model (extended variant)l - sequence context event classification
	Control		Tested								
	Digest/MSAP profile										
	SbfI /HpaII	SbfI /MspI	SbfI /HpaII	SbfI /MspI	Control (C)	Stressed (S)	C -> S transition				
‘0000	0	0	0	0	5′-^{m}C^{m}CGG-3′	5′-^{m}C^{m}CGG-3′	MM/MM->MM/MM	MP	Type IV - Type IV	MPex x 2; MPi x 2	
					3′-GGC^{m}C^{m}-5′	3′-GGC^{m}C^{m}-5′	MM/MM->M-/-M			MPex x 2; DMin x 2	DM-CG x 2
					5′-^{m}CCGG-3′	5′-^{m}CCGG-3′	M-/-M->M-/-M			MPex x 2; NMPin x 2	
					3′-GGCC^{m}-5′	3′-GGCC^{m}-5′	M-/-M->MM/MM			MPex x 2; DNin x 2	DN-CG x 2
‘0001	0	0	0	1	5′-^{m}C^{m}CGG-3′	5′-C^{m}CGG-3′	MM/MM->-M/--	DM	Type IV - Type III	DMex x 2; DMin; MPin	DM-CHG x 2; DM-CG
					3′-GGC^{m}C^{m}-5′	3′-GGCC-5′	MM/MM->-M/M-			DMex x 2; MPin x 2	DM-CHG x 2;
					5′-^{m}CCGG-3′	5′-C^{m}CGG-3′	M-/-M->-M/--			DMex x 2; DNin ; NMPin	DM-CHG x 2; DN-CG
					3′-GGCC^{m}-5′	3′-GGC^{m}C-5′	M-/-M->-M/M-			DMex x 2; DNin x 2	DM-CHG x 2; DN-CG x 2
‘0010	0	0	1	0	5′-^{m}C^{m}CGG-3′<br>3′-GGC^{m}C^{m}-5′	5′-^{m}CCGG-3′	MM/MM ->M-/--	DM, MP	Type IV - Type II	DMex; DMin x 2; MPex	DM-CHG; DM-CG x 2
					5′-^{m}CCGG-3′<br>3′-GGCC^{m}-5′	3′-GGCC-5′	M-/-M->M-/--			DMex; NMPin x 2; MPex	DM-CHG
‘0011	0	0	1	1	5′-^{m}C^{m}CGG-3′<br>3′-GGC^{m}C^{m}-5′	5′-CCGG-3′	MM/MM->--/--	DM	Type IV - Type I	DMex x 2; DMin x 2	DM-CHG x 2; DM-CG x 2

					5′-^{m}CCGG-3′						
						3′-GGCC-5′	M-/-M->--/--			DMex x 2; NMPin x 2	DM-CHG x 2
					3′-GGCC^{m}-5′						
					5′-C^{m}CGG-3′	5′-^{m}C^{m}CGG-3′	-M/-- ->MM/MM			DNex x 2; DNin; MPin	DN-CHG x 2; DN-CG
					3′-GGCC-5′	3′-GGC^{m}C^{m}-5′	-M/-- ->M-/-M			DNex x 2; DMin; NMPin	DN-CHG x 2; DM-CG
‘0100	0	1	0	0				DN	Type III - Type IV		
					5′-C^{m}CGG-3′	5′-^{m}CCGG-3′	-M/M- ->MM/MM			DNex x 2; MPin x 2	DN-CHG x 2
					3′-GGC^{m}C-5′	3′-GGCC^{m}-5′	-M/M- ->M-/-M			DNex x 2; DMin x 2	DN-CHG x 2; DM-CG x 2
					5′-C^{m}CGG-3′	5′-C^{m}CGG-3′	-M/-- -> -M/--			NMPex x 2; MPin; NMPin	
					3′-GGCC-5′	3′-GGCC-5′	-M/-- -> -M/M-			NMPex x 2; MPin; DNin	DN-CG
‘0101	0	1	0	1				NMP, MP	Type III - Type III		
					5′-C^{m}CGG-3′	5′-C^{m}CGG-3′	-M/M- -> -M/--			NMPex x 2; MPin; DMin	DM-CG
					3′-GGC^{m}C-5′	3′-GGC^{m}C-5′	-M/M- -> -M/M-			NMPex x 2; MPin x 2	
					5′-C^{m}CGG-3′	5′-^{m}CCGG-3′	-M/-- -> M-/--			DNex; DMin; NMPex ; NMPin	DN-CHG; DM-CG
					3′-GGCC-5′						
‘0110	0	1	1	0				DN, DM, NMP	Type III - Type II		
					5′-C^{m}CGG-3′	3′-GGCC-5′	-M/M- -> M-/--			DNex; DMin x 2; NMPex	DN-CHG; DM-CG x 2
					3′-GGC^{m}C-5′						
					5′-C^{m}CGG-3′	5′-CCGG-3′	-M/-- -> --/--			NMPex x 2; DMin; NMPin	DM-CG
					3′-GGCC-5′						
‘0111	0	1	1	1				NMP, DM	Type III - Type I		
					5′-C^{m}CGG-3′	3′-GGCC-5′	-M/M- -> --/--			NMPex x 2; DMin x 2	DM-CG x 2
					3′-GGC^{m}C-5′						
						5′-^{m}C^{m}CGG-3′					
1000	1	0	0	0	5′-^{m}CCGG-3′		M-/-- -> MM/MM	MP, DN	Type II - Type IV	MPex; DNex; DNin x 2	DN-CHG; DN-CG x 2
						3′-GGC^{m}C^{m}-5′					

					3′-GGCC-5′	5′-^{m}CCGG-3′<br>3′-GGCC^{m}-5′	M-/-- -> M-/-M			MPex; DNex; NMP x 2	DN-CHG
1001	1	0	0	1	5′-^{m}CCGG-3′	5′-C^{m}CGG-3′<br>3′-GGCC-5′	M-/-- -> -M/--	DN, DM, NMP	Type II - Type III	DMex; DNin; NMPex; NMPin	DM-CHG; DN-CG
					3′-GGCC-5′	5′-C^{m}CGG-3′<br>3′-GGC^{m}C-5′	M-/-- -> -M/M-			DMex; DNin; NMPex; DNin	DM-CHG; DN-CG x 2
1010	1	0	1	0	5′-^{m}CCGG-3′<br>3′-GGCC-5′	5′-^{m}CCGG-3′<br>3′-GGCC-5′	M-/-- -> M-/--	MP, NMP	Type II - Type II	MPex; NMPin x 2; NMPex	
1011	1	0	1	1	5′-^{m}CCGG-3′<br>3′-GGCC-5′	5′-CCGG-3′<br>3′-GGCC-5′	M-/-- -> --/--	DM, NMP	Type II - Type I	DMex; NMPin x 2; NMPin	DM-CHG
1100	1	1	0	0	5′-CCGG-3′	5′-^{m}C^{m}CGG-3′<br>3′-GGC^{m}C^{m}-5′	--/-- -> MM/MM	DN	Type I - Type IV	DNex x 2; DNin x 2	DN-CHG x 2; DN-CG x 2
					3′-GGCC-5′	5′-^{m}CCGG-3′<br>3′-GGCCm-5′	--/-- -> M-/-M			DNex x 2; NMPin x 2	DN-CHG x 2
1101	1	1	0	1	5′-CCGG-3′	5′-C^{m}CGG-3′<br>3′-GGCC-5′	--/-- -> -M/--	DN	Type I - Type III	NMPex x 2; DNin; NMPin	DN-CG
					3′-GGCC-5′	5′-C^{m}CGG-3′<br>3′-GGC^{m}C-5′	--/-- -> -M/M-			NMPex x 2; DNin x 2	DN-CG x 2
1110	1	1	1	0	5′-CCGG-3′<br>3′-GGCC-5′	5′-^{m}CCGG-3′<br>3′-GGCC-5′	--/-- -> M-/--	NMP, DN	Type I - Type II	NMPin x 2; NMPex; DNex;	DN-CHG
1111	1	1	1	1	5′-CCGG-3′<br>3′-GGCC-5′	5′-CCGG-3′<br>3′-GGCC-5′	--/-- -> --/--	NMP	Type I - Type I	NMPex x 2; NMPin x 2	

Table S1. The explanation of methylation change/status affecting restriction sites utilizing the General (basic and extended variants) and the Common model. Based on the MSAP-based approach, i.e., a four-digit code '0010 is evaluated when control (unstressed) materials treated with *SbfI*/*Hpa*II and *SbfI*/*Msp*I endonucleases cannot be digested due to the presence of restriction site methylation that blocs hydrolysis (mutations are not considered as a putative source of variation). However, when the same lines are stressed, and their DNAs are digested with the same enzyme pairs, a marker sequence is detected in the case of the *SbfI*/*Hpa*II digests. The interpretation of such a result based on site restriction methylation is that the site is fully methylated in the control line, whereas Al-treatment leads to partial demethylation. It is reflected as MM/MM ->M-/--, (where MM/MM means that external and internal cytosines of the restriction site are methylated in control lines, whereas only a single external cytosine remains methylated in stressed line), leading to the DArTseqMet marker evaluation only in one of the platform. An alternative explanation is that one external and one internal cytosine of controls is non-methylated, whereas, in Al-treated materials, only one external cytosine remains methylated. Thus, the '0010 code reflects DNA demethylation and partial methylation preservation status due to Al-treatment compared to the control. In the MSAP-based approach, such a code is classified as transitioning from Type IV to Type II (Fulneček and Kovařík, 2014; Wang et al., 2019).

When switching to the semi-quantitative MSAP-based (basic) model, additional information is gained. Then, the code is interpreted as DNA demethylation of one external cytosine, DNA demethylation of two internal cytosines present in the restriction site and preservation of the methylation status of external cytosine. Furthermore, an alternative explanation is also possible. According to it, a single external cytosine is demethylated due to Al-treatment; there are two non-methylated internal cytosines, and one external cytosine remains methylated. The other codes could be interpreted following similar analyses, resulting in a more sophisticated quantification analysis described for the semi-quantitative MSAP-based basic model. In its extended version, it is possible to get detailed information on symmetric sequence contexts by analyzing putative sequence contexts in the restriction site. Here, '0010 reflects DNA demethylation in the CHG context and DNA demethylation of two CG contexts. The other alternative is that DNA demethylation is within a single CHG context of the site. Following the reasoning described earlier (Bednarek et al., 2017), semi-quantitative MSAP-based events could be quantified and normalized.
