# Supplementary material for: DNA Methylation Changes Reflect Aluminum Stress in Triticale and Epigenetic Control of the Trait
Source: Int J Mol Sci. 2025 May 22;26(11):4995. doi: 10.3390/ijms26114995 (PMC12154425; doi:10.3390/ijms26114995)
Supplement: Supplementary file 1 [file ijms-26-04995-s001.zip › ijms-3615265-supplementary/Table S/Table S2.pdf]

Table S2. Pearson's correlation coefficients evaluated based on DArTseqMet NGS DNA markers utilizing the MSAP quantitative characteristics.

Variables	DM-c	DM-CHG	DM-b	DM-CG	NMP-c	NMP-b	MP-b	MP-c	M-b	NM-b	M-c	NM-c	DN-CG	DNM-b	DNM-c	DN-CHG
DM-c	**1**	**0.978**	**0.973**	**0.934**	**-0.873**	**-0.783**	0.439	0.169	0.367	-0.367	0.270	-0.270	0.097	0.095	0.077	0.090
DM-CHG	**0.978**	**1**	**0.991**	**0.946**	**-0.877**	**-0.807**	**0.480**	0.282	0.389	-0.389	0.328	-0.328	0.093	0.082	0.069	0.068
DM-b	**0.973**	**0.991**	**1**	**0.980**	**-0.913**	**-0.850**	**0.547**	0.366	0.453	-0.453	0.397	-0.397	0.122	0.110	0.080	0.093
DM-CG	**0.934**	**0.946**	**0.980**	**1**	**-0.937**	**-0.886**	**0.629**	**0.481**	**0.534**	**-0.534**	**0.489**	**-0.489**	0.160	0.147	0.094	0.126
NMP-c	**-0.873**	**-0.877**	**-0.913**	**-0.937**	**1**	**0.974**	**-0.683**	**-0.503**	**-0.749**	**0.749**	**-0.691**	**0.691**	-0.451	-0.449	-0.363	-0.414
NMP-b	**-0.783**	**-0.807**	**-0.850**	**-0.886**	**0.974**	**1**	**-0.803**	**-0.621**	**-0.855**	**0.855**	**-0.765**	**0.765**	**-0.502**	**-0.485**	-0.363	-0.430
MP-b	0.439	**0.480**	**0.547**	**0.629**	**-0.683**	**-0.803**	**1**	**0.808**	**0.819**	**-0.819**	**0.624**	**-0.624**	0.240	0.181	-0.030	0.105
MP-c	0.169	0.282	0.366	**0.481**	**-0.503**	**-0.621**	**0.808**	**1**	**0.690**	**-0.690**	**0.746**	**-0.746**	0.276	0.195	0.074	0.093
M-b	0.367	0.389	0.453	**0.534**	**-0.749**	**-0.855**	**0.819**	**0.690**	**1**	**-1.000**	**0.904**	**-0.904**	**0.729**	**0.712**	**0.535**	**0.636**
NM-b	-0.367	-0.389	-0.453	**-0.534**	**0.749**	**0.855**	**-0.819**	**-0.690**	**-1.000**	**1**	**-0.904**	**0.904**	**-0.729**	**-0.712**	**-0.535**	**-0.636**
M-c	0.270	0.328	0.397	**0.489**	**-0.691**	**-0.765**	**0.624**	**0.746**	**0.904**	**-0.904**	**1**	**-1.000**	**0.813**	**0.785**	**0.697**	**0.691**
NM-c	-0.270	-0.328	-0.397	**-0.489**	**0.691**	**0.765**	**-0.624**	**-0.746**	**-0.904**	**0.904**	**-1.000**	**1**	**-0.813**	**-0.785**	**-0.697**	**-0.691**
DN-CG	0.097	0.093	0.122	0.160	-0.451	**-0.502**	0.240	0.276	**0.729**	**-0.729**	**0.813**	**-0.813**	**1**	**0.957**	**0.943**	**0.841**
DNM-b	0.095	0.082	0.110	0.147	-0.449	**-0.485**	0.181	0.195	**0.712**	**-0.712**	**0.785**	**-0.785**	**0.957**	**1**	**0.955**	**0.962**
DNM-c	0.077	0.069	0.080	0.094	-0.363	-0.363	-0.030	0.074	**0.535**	**-0.535**	**0.697**	**-0.697**	**0.943**	**0.955**	**1**	**0.892**
DN-CHG	0.090	0.068	0.093	0.126	-0.414	-0.430	0.105	0.093	**0.636**	**-0.636**	**0.691**	**-0.691**	**0.841**	**0.962**	**0.892**	**1**

c, b, and e - state for the Common, basic and extended variants of the General models. Values in bold are different from 0 with a significance level alpha=0.05
